# UCH-L1 inhibitor LDN-57444 hampers mouse oocyte maturation by regulating oxidative stress and mitochondrial function and reducing ERK1/2 expression

**DOI:** 10.1042/BSR20201308

**Published:** 2020-10-30

**Authors:** Pan Yuan, Li Zhou, Xiaona Zhang, Lan Yao, Jun Ning, Xiao Han, Caifeng Ming, Yunhe Zhao, Liqun Zhang

**Affiliations:** 1 Center for Prenatal Diagnosis of Reproductive Medicine, The First Hospital of Jilin University, Changchun 130021, China; 2 Department of Gynaecology and Obstetrics, The First Hospital of Jilin University, Changchun 130021, China; 3 Department of Anesthesiology, The First Hospital of Jilin University, Changchun 130021, China

**Keywords:** maturation, mouse, oocytes, oxidative stress, spindle body, UCH-L1

## Abstract

Oocyte maturation is a prerequisite for successful fertilization and embryo development. Incomplete oocyte maturation can result in infertility. Ubiquitin carboxy-terminal hydrolase L1 (UCH-L1) has been found to be implicated in oocyte maturation and embryo development. However, the cellular and molecular mechanisms of UCH-L1 underlying oocyte maturation have not been fully elucidated. In the present study, we observed that the introduction of UCH-L1 inhibitor LDN-57444 suppressed first polar body extrusion during mouse oocyte maturation. The inhibition of UCH-L1 by LDN-57444 led to the notable increase in reactive oxygen species (ROS) level, conspicuous reduction in glutathione (GSH) content and mitochondrial membrane potential (MMP), and blockade of spindle body formation. As a conclusion, UCH-L1 inhibitor LDN-57444 suppressed mouse oocyte maturation by improving oxidative stress, attenuating mitochondrial function, curbing spindle body formation and down-regulating extracellular signal-related kinases (ERK1/2) expression, providing a deep insight into the cellular and molecular basis of UCH-L1 during mouse oocyte maturation.

## Introduction

Oocytes, female gametocytes or germ cells involved in reproduction, can transmit maternal nuclear and mitochondrial genome information to the embryo and determine embryo developmental potential in women [1]. Oocyte maturation is a physiological event ahead of successful fertilization and embryo development, and failure of or incomplete oocyte maturation can bring about infertility [2]. Most mammalian oocytes are usually arrested at the diplotene stage of prophase I (germinal vesicle (GV) stage) prior to ovulation. During each reproductive cycle, preovulatory luteinizing hormone outpouring induces meiosis resumption and progression till oocyte maturation (metaphase II (MII) stage). After meiosis resumption, oocytes undergo a sequential series of changes, including GV breakdown (GVBD), chromosome condensation, spindle formation and first polar body extrusion [2,3]. *In vitro* maturation (IVM) of immature oocytes, a vital technique in fertility preservation, has attracted much attention from researchers in recent years due to its specific advantages [4,5]. For example, this technique requires no or little gonadotropin supplementation *in vivo*, which can significantly reduce ovarian hyperstimulation syndrome risk, medical expenses and drug-related side effects [5,6]. Despite the great advances in oocyte IVM technique, there are still a lot of controversial issues that need to be addressed [7]. An in-depth understanding on cellular and molecular basis of oocyte IVM contributes to fertility protection, pregnancy rate improvement and the better management of ovarian hyperstimulation syndrome and infertility.

Mitochondria, cellular organelles responsible for ATP synthesis, play vital roles in various biological processes such as metabolism, calcium homeostasis and cell apoptosis [8]. Mitochondria dysfunction can influence oocyte quality, maturation and fertilization, resulting in the deterioration of reproductive outcomes. Moreover, oocyte mitochondria are the major energy suppliers in the process of preimplantation embryonic development [9]. Furthermore, some studies pointed out that mature oocytes with reduced mitochondrial membrane potential (MMP) had limited sperm penetrability and impaired fertilization competence [10–12]. In addition, mitochondria are the major sites of reactive oxygen species (ROS) production [13] and mitochondrial dysfunction is closely related with the dynamic change of ROS level in oocytes [12]. Additionally, previous studies showed that ROS contributed to oocyte maturation under physiological conditions, while ROS over-production or antioxidant loss could influence oocyte quality and developmental competence, giving rise to anovulation [14]. For instance, a moderate elevation of ROS level contributed to meiotic resumption from diplotene and M-II arrest phases, and excessive ROS generation blocked meiotic cell cycle progression and induced cell apoptosis in oocytes [15,16]. Recently, a growing body of evidence suggests that oocyte mitochondria function improvement and mitochondria replacement might can be used for elevating reproductive capability and treating for mitochondrial diseases [9,12].

Ubiquitin carboxy-terminal hydrolase L1 (UCH-L1), also named as pGp 9.5, can function as a deubiquitinating enzyme in monomeric form and an ubiquitin ligase in dimer or oligomeric form [17]. Abnormal expression of UCH-L1 has been reported to be implicated in the pathogenesis of multiple diseases such as cancers [18], nervous system diseases [19] and lung injury [20]. Moreover, previous studies showed that UCH-L1 was selectively and abundantly expressed in multiple reproductive cells such as oocytes, spermatogonia and testis cells [21]. In addition, UCH-L1 has been found to be involved in the regulation of oocyte maturation and embryo development [22,23]. However, the molecular mechanisms of UCH-L1 underlying oocyte maturation have not been fully elucidated.

## Methods

### Oocyte preparation and IVM

All mouse procedures were performed in Jilin University and approved by Animal Care and Use Committee of Jilin University. The experiments were conducted in accordance with the institutional guidelines of the National Institutes of Health Guide for the Care and Use of Laboratory Animals.

ICR female mice (7-week-old, 20–30 g) were purchased from Laboratory Animal Center of Zhengzhou University (Zhengzhou, China) and fed for ∼1 week under a standard condition to allow them to acclimatize. Then, the mice were superovulated by the intraperitoneal injection of 5 IU of pregnant mares’ serum gonadotrophin (PMSG; Amyjet Scientific, Beijing, China). After 48 h, mice were killed using intraperitoneal phenobarbital injection (150 mg/kg; Guangdong Bangmin Pharmaceutical Co., Ltd.) and ovaries were removed.

After the removal of excessive tissues and fat under a microscope using the tweezers, oocytes were isolated from ovaries through vigorous poking using a disposable syringe containing an 18G needle. Oocytes (diameter: 80–100 μm, round) with an intact and compact cumulus, GV structure in the center and thick zona pellucida were maintained in drops of M16 medium (Sigma–Aldrich, St. Louis, MO, U.S.A.) covered with mineral oil in a humidified atmosphere containing 5% CO_2_ at 37°C. To explore the effect of UCH-L1 inhibition on mouse oocyte maturation, 20 µM of LDN-57444 (Selleck, Houston, Texas, U.S.A.) was added into M16 medium as previously described [21]. Then, first polar body extrusion rate was measured at 18 h after LDN-57444 or DMSO treatment.

### ROS level determination

ROS level was measured using the 6-carboxy-2′,7′-dichlorodihydrofluorescein diacetate (DCHFDA, Thermo Scientific, Rockford, IL, U.S.A.) following the protocols of manufacturer. Briefly, oocytes were incubated with 10 mM of DCHFDA for 30 min at 37°C. Next, samples were imaged with a fluorescent microscope and the fluorescence intensity was quantified using the ImageJ software.

### Glutathione level determination

Glutathione (GSH) level was determined using 10 mM of CellTracker™ Blue CMF2HC Dye (4-chloromethyl-6,8-difluoro-7-hydroxycoumarin) (Thermo Scientific) in oocytes at 18 h after DMSO or LDN-57444 treatment. Samples were imaged with a fluorescent microscope and the fluorescence intensity was quantified using the ImageJ software.

### MMP analysis

After three washes with PBS containing 1% polyvinyl alcohol (PBS-PVA), mature oocytes were incubated with 2 mM of tetraethylbenzimidazolylcarbocyanine iodide (JC-1) fluorescent probe (Thermo Scientific) in PBS-PVA solution for 30 min at 37°C. Next, Red and Green fluorescence intensity was rescored using a fluorescent microscope and quantified through ImageJ software after three washes with PBS-PVA. Finally, the ratio of Red/Green fluorescence intensity was calculated to determinate MMP changes with high value of Red/Green fluorescence intensity ratio as the indicator of MMP increase.

### Spindle abnormality detection

After washing thrice with PBS-PVA solution, matured oocytes were fixed with 3.7% paraformaldehyde (diluted in PBS-PVA solution) for 30 min and permeablized for 15 min at 37°C using the 1% Triton X-100 solution. After blocking with PBS-PVA blocking buffer containing 1% BSA for 1 h at room temperature, these oocytes were incubated overnight with FITC-conjugated α-tubulin antibody (Abcam, Cambridge, U.K.) in blocking buffer. Next, these oocytes were stained with 10 μg/ml of Hoechst 33342 (blue) in PBS to visualize DNA. After three washes with PBS-PVA buffer, samples were mounted on microscope slide and imaged using a Confocal Laser-scanning Microscope (Zeiss LSM 700 META, Jena, Germany). Finally, the intensity of fluorescence signals was analyzed using ImageJ software.

### Western blot assay

At 18 h after DMSO or LDN-57444 treatment, total proteins were extracted from oocytes using the RIPA lysis buffer (Beyotime, Shanghai, China) containing protease inhibitors (Thermo Scientific, Waltham, MA, U.S.A.) and quantified using the Pierce™ BCA Protein Assay Kit (Thermo Scientific). Next, an equal amount of proteins (30 μg/lane) were separated through 10% sodium dodecyl sulfate/polyacrylamide gel electrophoresis (SDS/PAGE) and transferred to polyvinylidene fluoride (PVDF) membranes (Millipore, Bedford, MA, U.S.A.). Subsequently, the membranes were blocked for 1 h at room temperature with 5% skim milk and probed overnight at 4°C with primary antibodies against UCH-L1 (#13179, Cell Signaling Technology, Danvers, MA, U.S.A.), extracellular signal-related kinases (ERK1/2; ab17942, Abcam) and glyceraldehyde phosphate dehydrogenase (GAPDH) (ab181602, Abcam). After incubating for 1 h at room temperature with horseradish peroxidase (HRP) conjugated goat anti-rabbit secondary antibody (ab205718, Abcam), the membranes were exposed with Pierce™ ECL Western Blotting Substrate (Thermo Fisher Scientific). Finally, the intensity of protein signals was estimated using the Quantity One software Version 4.1.1 (Bio-Rad Laboratories, Hercules, CA, U.S.A.).

### Reverse transcription-polymerase chain reaction (RT-qPCR) assay

Total RNA was extracted from oocytes using TRIzol reagent (Thermo Scientific) following the manufacturer’s instructions. RNA was reversely transcribed into cDNA using SuperScript III reverse transcriptase (Thermo Scientific). Quantitative PCR analysis was performed using SYBR™ Green PCR Master Mix (Thermo Scientific) and PCR primers on ABI 7500 Real-Time PCR System (Thermo Scientific). Primer sequences were presented as follows: 5′-CTTCAACCCAAACAAGCGCA-3′ (forward) and 5′-CCATGTCGAAGGTGAATGGC-3′ (reverse) for ERK1; 5′-CCAACCTCTCGTACATCGGA-3′ (forward) and 5′-ATGGTTGGTGCCCGGATG-3′ (reverse) for ERK2 and 5′-TGTTACCAACTGGGACGACGACA-3′ (forward) and 5′-CTGGGTCATCTTTTCACGGT-3′ (reverse) for β-actin. β-actin functioned as the internal control to normalize the expression of ERK1/2.

### LDN-57444 proteasome activity detection

Proteasome activity is measured using the Proteasome Activity Assay Kit (Abcam, CA, U.S.A.), which principally measures the intensity of highly fluorescent 7-amido-4-methylcoumarin (AMC) released from AMC-tagged peptide substrate (Succ-LLVY-AMC in DMSO) in the presence of proteolytic activity. Briefly, standard curve was prepared using AMC Standard. At 18 h after LDN-57444 treatment, cells were collected and resuspended in 0.5% NP-40 solution. After high-speed centrifugation, cell supernatant was co-incubated with AMC-tagged peptide substrate in the presence or absence of proteasome inhibitor in the dark. Fluorescence was measured at excitation (Ex) wavelength/emission (Em) wavelength = 350/440 nm at 0 and 0.5 h after incubation.

### Statistical analysis

All experiments were repeated at least three times and results were presented as means ± SEMs. Difference analysis between groups was conducted using unpaired *t* test with *P*<0.05 as statistically significant. Difference analysis among groups was performed using one-way ANOVA and Tukey’s test.

## Results

### The introduction of UCH-L1 inhibitor LDN-57444 markedly suppressed mouse oocyte maturation

**Figure 1:**
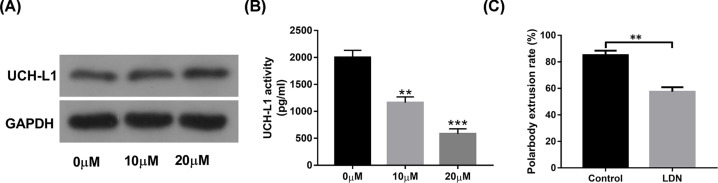
The introduction of UCH-L1 inhibitor LDN-57444 markedly reduced proteasome activity and inhibited mouse oocyte maturation Mouse oocytes were matured for 18 h in the presence of DMSO (control) or LDN-57444 (10, 20 µM), followed by the measurement of UCH-L1 protein level (**A**), proteasome activity (**B**), first polar body extrusion rate (**C**). **P<0.01. ***P<0.001.

First, our data revealed that the introduction of LDN-57444 had not much influence on UCH-L1 expression in oocytes (Figure 1A). Since UCH-L1 is a component of ubiquitin proteasome system, the effect of LDN-57444 on proteasome activity was further measured in mouse oocytes. Our outcomes showed that the addition of LDN-57444 led to the notable reduction in proteasome activity (Figure 1B). To explore the cellular and molecular basis of UCH-L1 underlying oocyte maturation, 20 µM of UCH-L1 inhibitor LDN-57444 was used in oocytes as previously described [21,23,24]. Our data revealed that first polar body extrusion rate was notably reduced in LDN-57444-treated oocytes (extrusion rate: 57.47%, *n*=98) than that in control group (extrusion rate: 85.01%, *n*=97) (Figure 1C), suggesting that the inhibition of UCH-L1 proteasome activity by LDN-57444 hindered mouse oocyte maturation.

### The introduction of UCH-L1 inhibitor LDN-57444 induced ROS excessive accumulation and caused cellular GSH level reduction in MII phase oocytes

**Figure 2:**
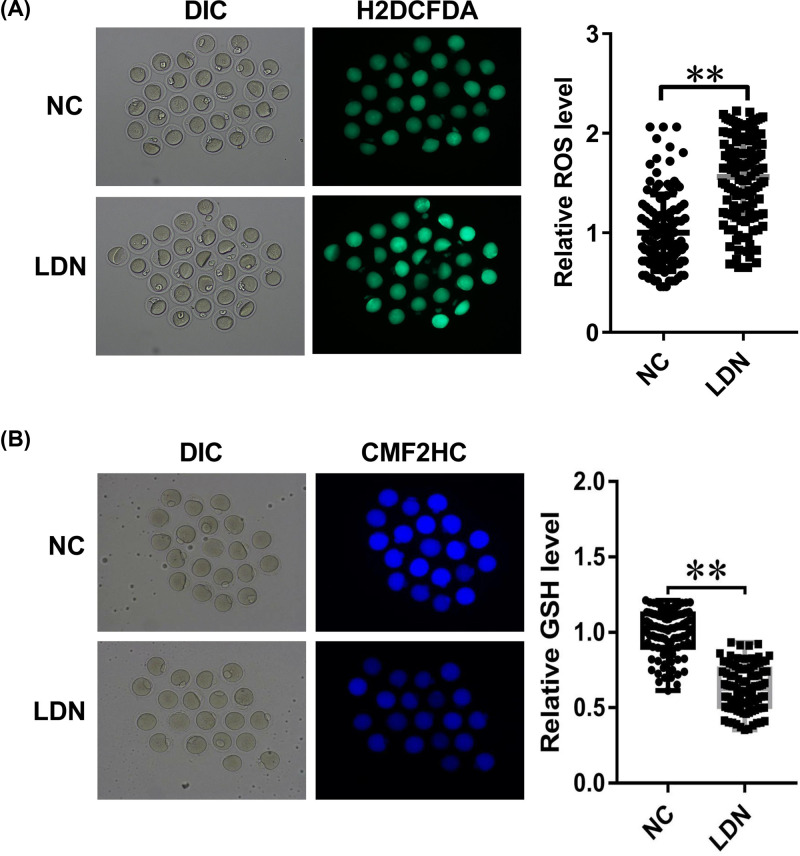
The introduction of UCH-L1 inhibitor LDN-57444 increased oxidative stress during mouse oocyte maturation (**A,B**) Mouse oocytes were matured with DMSO or LDN-57444 (20 µM). Eighteen hours later, ROS (A) and GSH (B) levels were determined through corresponding fluorescence probes. **P<0.01.

Next, the effect of LDN-57444 on oxidative stress was examined through measuring ROS and GSH levels in MII phase oocytes. H2DCFDA (2′,7′-dichlorodihydrofluorescein diacetate) was a cell-permeable probe used to detect intracellular ROS). After diffusing into cells, H2DCFDA is deacetylated by cellular esterases to a non-fluorescent compound, which is later oxidized by ROS into 2′,7′-dichlorofluorescein (DCF). DCF is highly fluorescent and is detected by fluorescence spectroscopy with excitation/emission at 495/525 nm. The intensity of green fluorescence was in proportion to ROS level. Our data revealed that ROS level was remarkably increased in LDN-57444 treatment group (*n*=111) relative to control group (*n*=110) (Figure 2A). CellTracker Blue CMF2HC is a fluorescent dye that can freely pass through cell membranes into cells, where it is transformed into cell-impermeant reaction products. In eukaryotic cells, GSH is the most abundant thiol. CMF2HC contains a chloromethyl group that can react with thiol groups, utilizing a glutathione S-transferase-mediated reaction. Hence, the fluorescent intensity of CMF2HC can reflect GSH cellular level. As presented in Figure 2B, GSH level was notably reduced in LDN-57444-stimulated oocytes (*n*=75) versus control group (*n*=74). These outcomes suggested that the inhibition of UCH-L1 by LDN-57444 increased oxidative stress during mouse oocyte maturation.

### The introduction of UCH-L1 inhibitor LDN-57444 impaired cellular mitochondria function in MII phase oocytes

MMP has been identified as the crucial indicator of mitochondrial function and cell health. The loss of MMP is a hallmark for apoptosis. It is an early event preceding phosphatidylserine externalization and coinciding with caspase activation.

JC-1 is a cell membrane permeable fluorescent lipophilic carbocyanine dye used to measure MMP. In normal non-apoptotic cells, JC-1 accumulates as aggregates in the mitochondrial in response to high MMP, yielding a red to orange fluorescence. The brightness of red fluorescence is proportional to MMP and varies among different cell types. However, in apoptotic and necrotic cells, which have diminished MMP, JC-1 predominantly exists in the monomer form that yields green fluorescence. Hence, JC-1 Red/Green fluorescence ratio can reflect the level of MMP.

**Figure 3:**
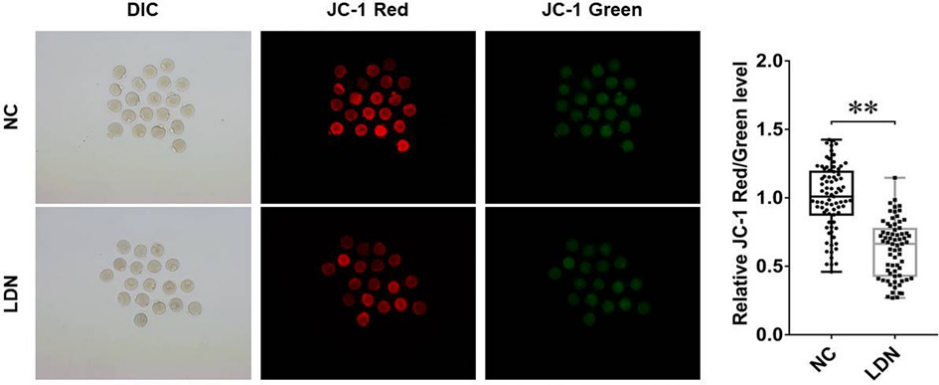
The introduction of UCH-L1 inhibitor LDN-57444 impaired cellular mitochondria function in MII phase oocytes Mouse oocytes were matured with DMSO or LDN-57444 (20 µM) for 18 h, followed by the measurement of JC-1 (Red/Green) fluorescence intensity. **P<0.01.

Our study demonstrated that the introduction of UCH-L1 inhibitor LDN-57444 led to the conspicuous reduction in MMP in MII phase oocytes (Figure 3), as evidenced by the reduction in JC-1 Red/Green fluorescence ratio in LDN-57444 treatment group (*n*=69) versus control group (*n*=79), hinting the detrimental effect of UCH-L1 loss on mitochondria function during oocyte maturation.

### The introduction of UCH-L1 inhibitor LDN-57444 blocked spindle body formation during mouse oocyte maturation

**Figure 4:**
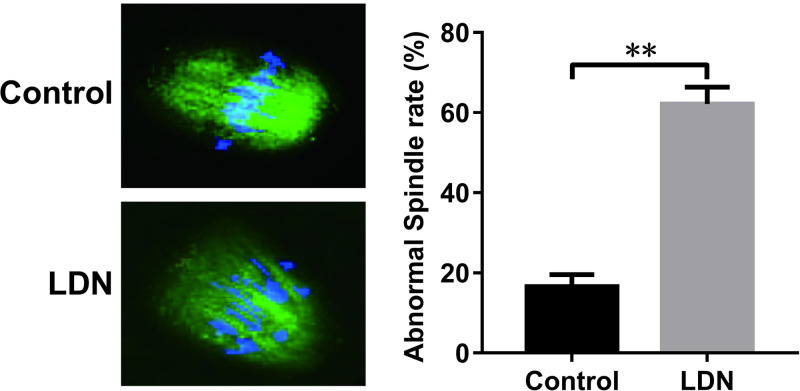
The introduction of UCH-L1 inhibitor LDN-57444 blocked spindle body formation during mouse oocyte maturation Mouse oocytes were matured with DMSO or LDN-57444 (20 µM). After 18 h, oocytes were fixed, incubated with FITC-conjugated α-tubulin antibody, stained with Hoechst 33342 solution and analyzed by confocal microscopy. Green, α-tubulin; blue, Hoechst 33342 for DNA staining. **P<0.01.

α-tubulin, an indispensable constituent of microtubule and spindle body, has been well known as a crucial player in the formation of spindle body. Hence, FITC-conjugated α-tubulin antibody was used to examine whether there were some alteration to spindle body structure in the presence or absence of LDN-57444. As displayed in Figure 4, a higher percentage of microtubule and chromosome misarrangement was observed in LDN-57444-treated oocytes (62.18%, *n*=40) compared with control group (16.56%, *n*=42), suggesting that the introduction of LDN-57444 blocked spindle body formation during mouse oocyte maturation.

### The introduction of UCH-L1 inhibitor LDN-57444 reduced the expression of ERK1/2 protein levels in MII phase oocytes

**Figure 5:**
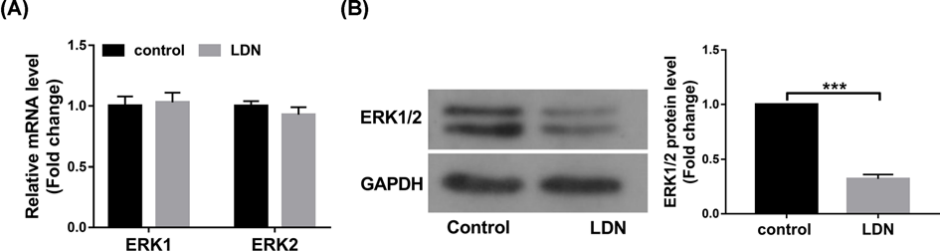
The introduction of UCH-L1 inhibitor LDN-57444 reduced the expression of ERK1/2 protein levels in MII phase oocytes (**A,B**) Mouse oocytes were matured with DMSO or LDN-57444 (20 µM) for 18 h. Then, ERK1/2 mRNA (**A**) and protein (B) levels were measured through RT-qPCR and Western blot assays, respectively. ***P<0.001.

Next, we demonstrated that the introduction of LDN-57444 had not much influence on ERK1 and ERK2 mRNA levels in MII phase oocytes (Figure 5A). However, a notable reduction in ERK1/2 protein levels was observed in MII phase oocytes after the stimulation of UCH-L1 inhibitor LDN-57444 (Figure 5B).

## Discussion

In the present study, we demonstrated that the introduction of UCH-L1 inhibitor LDN-57444 suppressed mouse oocyte maturation by improving oxidative stress, inducing mitochondrial dysfunction, curbing spindle body formation, and reducing ERK1/2 protein expression.

First, our data revealed that UCH-L1 inhibitor LDN-57444 did not influence UCH-L1 protein expression, but could remarkably reduce UCH-L1 proteasome activity during mouse oocyte maturation. Moreover, the introduction of LDN-57444 led to the notable reduction in first polar body extrusion rate during mouse oocyte maturation, while polar body extrusion is an indicator of oocyte maturation. In other words, our outcomes showed that LDN-57444 inhibited mouse oocyte maturation. Consistent with our results, Susor et al. demonstrated that the inhibition of UCH-L1 by its inhibitor C30 hindered meiotic progression and curbed GVBD in oocytes cultured in maturation medium [22]. The inhibition of UCH-L1 hindered cortical granule (CG) migration, curbed meiotic spindle formation and reduced fertilization rate in porcine oocytes [23]. However, some studies pointed out that the introduction of UCH-L1 inhibitor C16 or C30 did not hamper meiotic progression of bovine oocytes, but induced the increase in polyspermy rate in bovine zygotes after *in vitro* fertilization [24]. Moreover, UCHL1 has been found to be involved in antipolyspermy defense *in vitro* in mouse [25] and pig [26]. In addition, the introduction of UCH-L1 inhibitor LDN-57444 led to the notable elevation in ployspermy and sperm–egg fusion events in zona pellucida-free eggs from superovulated CF-1 female mice [27].

ROS, including peroxides, superoxides, hydroxyl radical and singlet oxygen, can be generated during various biochemical reactions within cells and organelles (e.g. mitochondria and peroxisomes) [28,29]. Recently, ROS has been appreciated as a crucial player in multiple diseases (e.g. male infertility, cancers and neurodegenerative diseases) due to its regulatory effects on cell signaling proteins (e.g. NF-κB, mitogen-activated protein kinases (MAPKs) and PI3K-Akt), protein kinases and ubiquitination/proteasome system [30–33]. Moreover, ROS and antioxidants play vital roles in the development and maturation of oocytes [14,34]. Antioxidants, including superoxide dismutase (SOD), GSH, catalase and multiple peroxidases, can inhibit ROS over-production and protect oocytes from ROS-mediated detrimental effects in the reproductive system [14]. For instance, previous studies showed that ROS level and DNA damage degree were markedly increased in mouse oocytes exposed to follicular fluid from patients with endometriosis, and the increase in ROS level curbed oocyte maturation in endometriosis [35]. GSH mitigated cadmium-triggered porcine oocyte meiotic defects (e.g. the abnormality of spindle organization, chromosome alignment and actin polymerization, and disruption of mitochondrial integrity and cortical granules dynamics) through reducing ROS level, lessening DNA damage and inhibiting cell apoptosis [36]. Also, a prior document demonstrated that UCH-L1 overexpression facilitated cell invasion through increasing cellular ROS and H_2_O_2_ levels via deubiquitinating NADPH oxidase 4 (NOX4) in murine metastastic melanoma (B16F10) cells [37], suggesting the correlation of UCH-L1 and oxidative stress. Our data showed that the inhibition of UCH-L1 by LDN-57444 led to the substantial accumulation of ROS and remarkable reduction in GSH level in MII phase oocytes.

Next, we demonstrated that MMP was markedly reduced in MII phase oocytes following the introduction of UCH-L1 inhibitor LDN-5744, suggesting that the inhibition of UCH-L1 impaired mitochondrial function. Lee et al. demonstrated that the reduction in MMP by FCCP (carbonyl cyanide *p*-(tri-fluromethoxy) phenyl-hydrazone) curbed ATP generation and first polar body extrusion and blocked blastocyst formation with no effect on total cell number and apoptosis during porcine oocyte maturation [38]. Also, Ge et al. demonstrated that FCCP inhibited nuclear maturation, reduced the proportion of oocytes with normal spindle formation and chromosome alignment and evenly distributed mitochondria, and hampered blastocyst formation during mouse oocyte maturation [39].

In addition, our data showed that the inhibition of UCH-L1 led to the abnormal arrangement of α-tubulin and chromosome and hampered spindle body formation during mouse oocyte maturation. Consistently, Bheda et al. showed that UCH-L1 suppressed tubulin polymerization and microtubule formation in transformed cells during mitosis [40]. Kabuta et al. revealed that UCH-L1 could interact with tubulin, and UCH-L1 I93M mutant and carbonyl-modified UCH-L1 facilitated tubulin polymerization [41].

ERK1/2, members of MAPK superfamily, have been found to be closely associated with oocyte maturation [42,43]. For example, a previous study pointed out that ERK1/2 expression was notably reduced in matured bovine oocytes compared with unmatured or control oocytes [44]. ERK1/2 knockdown led to the disorganization of chromosome separation and abnormal extrusion of polar body 2 during mouse oocyte maturation [45]. In this text, we demonstrated that ERK1/2 protein levels were markedly down-regulated in MII phase oocytes following the introduction of UCH-L1 inhibitor LDN-57444.

Taken together, these data disclosed that the inhibition of UCH-L1 by LDN-57444 hampered mouse oocyte maturation by reducing ROS level and MMP, increasing GSH level, inhibiting spindle formation and reducing ERK1/2 protein level, deepening our understanding on cellular and molecular mechanisms of UCH-L1 underlying mouse oocyte maturation. Our data also suggested the potential values of UCH-L1 loss in reducing oocyte developmental competence, inducing female infertility and hampering embryo development. However, our experimental design was rough and our conclusion needs to be further validated by other experiments. Also, downstream signaling pathways and regulatory molecules require to be further explored.

## Data Availability

The data and material presented in this manuscript are available from the corresponding author on reasonable request.

## Abbreviations

CMF2HC: 4-chloromethyl-6,8-difluoro-7-hydroxycoumarin
DCF: 2′,7′-dichlorofluorescein
DCFHDA/H2DCFDA: 2′,7′-dichlorodihydrofluorescein diacetate
ERK1/2: extracellular signal-related kinase
FCCP: carbonyl cyanide *p*-(tri-fluromethoxy) phenyl-hydrazone
GAPDH: glyceraldehyde phosphate dehydrogenase
GSH: glutathione
GV: germinal vesicle
GVBD: GV breakdown
HRP: horseradish peroxidase
IVM: *in vitro* maturation
JC-1: tetraethylbenzimidazolylcarbocyanine iodide
MAPK: mitogen-activated protein kinase
MMP: mitochondrial membrane potential
MII: metaphase II
NOX4: NADPH oxidase 4
PVA: polyvinyl alcohol
PVDF: polyvinylidene fluoride
ROS: reactive oxygen species
RT-qPCR: reverse transcription-polymerase chain reaction
SDS/PAGE: sulfate/polyacrylamide gel electrophoresis
SOD: superoxide dismutase
UCH-L1: Ubiquitin carboxy-terminal hydrolase L1
